# Hairdye-Induced Hepatitis: An Unusal Cause of Acute Hepatitis

**DOI:** 10.4103/1319-3767.48999

**Published:** 2009-04

**Authors:** Varadaraj P. Gokak, Sasikala Mitnala, Ramji Cheemalkonda, Rupa Banarjee, Nagaraj Rao Padaki, D. Nageshwar Reddy

**Affiliations:** Department of Medical Gastroenterology, Asian Institute of Gastroenterology, Hyderabad, India. E-mail: aigindia@yahoo.co.in; 1 Department of Pathology, Asian Institute of Gastroenterology, Hyderabad, India; 2 Department of Hepatology, Asian Institute of Gastroenterology, Hyderabad, India

Sir,

We present an unusual case of acute hepatitis caused by hair dye.

Hair dye contains various mutagenic and carcinogenic chemicals and is examined as a risk factor for various malignancies. Hair dye-induced hepatitis is a rare condition. There is only one case of hair dye-induced hepatitis reported in the literature.[1]

A 33-year-old healthy young female presented with pruritus, skin lesions and jaundice 2 days after using a new hair dye rather than her usual one. Clinically, she was febrile, icteric, with hepatosplenomegaly but no ascites. Laboratory investigations showed hypereosinophilia of 20%, abnormal liver profile with total bilirubin of 13.5 mg/dL, conjugated 6.8 mg/dL, alanine transaminase 190 IU/L, aspartate transminase 152 IU/L, alkaline phosphatase of 227 IU/L and albumin being 3.7 g/dL. An ultrasound of the abdomen showed features of acute hepatitis and a normal biliary system. The prothrombin time was normal.

Work-up for acute hepatitis indicated negative IgM antibody for hepatites A, E, and anti-HBc antibody. Additionally, hepatitis B virus DNA was undetectable. Serology for cytomegalovirus, Herpes simplex virus, Epstein barr virus, leptospira and malaria were negative. Wilson's and autoimmune markers were also negative. Drug-induced hepatitis was considered in view of chronology of events. Because the patient had an allergic reaction, lymphocyte activation test was performed to study if the hair dye in question induced immune-mediated drug reaction leading to acute hepatitis.

**Figure 1:**
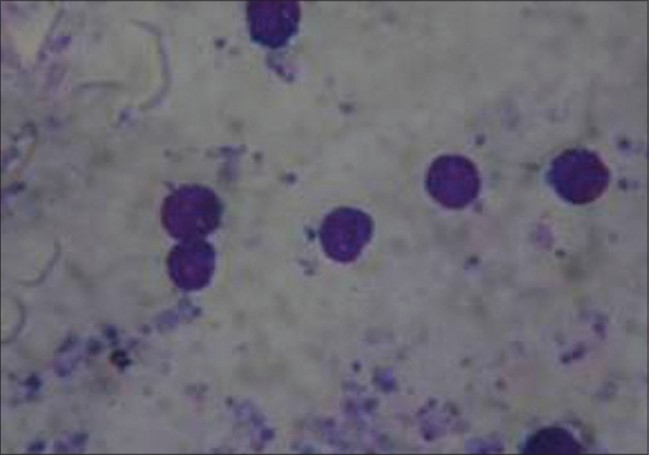
All reactive lymphocytes after 20 hours of incubation (oil immersion pictures)

Lymphocytes were isolated using Histopaque, 1077 (Sigma-Aldrich, St.louis, USA) by centrifugation.[2] Lymphocytes from a normal person and from a patient were subjected to hair dye exposure. Control was patient lymphocytes without hair dye. Suspension from the reaction mixture was taken out at different intervals and smears were examined for lymphocyte reactivity and adenosine deaminase (ADA) level represent lymphocyte activation. At the end of 60 min, 70% and at the second hour, 90% of the lymphocytes were activated as against the normal lymphocytes with no reactivity and only 3% of the controls. The ADA levels increased by 20% and 30% by the end of the first and second hour, respectively. Results obtained in this ***in vitro*** lymphocyte activity experiment strongly suggest that hair dye causes immune-mediated drug reaction in hypersensitive individuals [Figure 1].

Hair dye is a well-recognized cause of contact dermatitis. Routinely used hair dyes contain multiple components viz Toluene-2, 5-diamine, resorcin, cetearyl alcohol, polyethylene glycol, etc. Chemicals of the hair dye can be absorbed into the body through a wound, damaged skin or by aspiration of the spray during dyeing. The pathogenesis of drug-induced hepatotoxicity usually involves either the parent drug or its metabolite, which affects the hepatocytes directly or elicits an immune response.[3]

The chronological course of the present patient, allergic skin manifestations and ***in vitro*** lymphocyte reactivity to hair dye supported hair dye-induced immune-mediated hepatitis.[4] Liver biopsy was not performed as investigations supported diagnosis and her liver functions improved after 2 weeks of cessation of hair dye.

Thus, the present case suggests a possible role of hair dye in inducing an immune-mediated drug reaction in hypersensitive individuals and a lymphocyte activation test may be utilized to diagnose the immune-mediated drug-induced hepatotoxicity.
